# Imatinib binding to human c-Src is coupled to inter-domain allostery and suggests a novel kinase inhibition strategy

**DOI:** 10.1038/srep30832

**Published:** 2016-08-02

**Authors:** Yuko Tsutsui, Daniel Deredge, Patrick L. Wintrode, Franklin A. Hays

**Affiliations:** 1 Department of Biochemistry and Molecular Biology, University of Oklahoma Health Sciences Center, Oklahoma City, Oklahoma 73104, USA; 2 Department of Pharmaceutical Sciences, University of Maryland School of Pharmacy, Baltimore, Maryland 21201, USA; 3 Stephenson Cancer Center, University of Oklahoma Health Sciences Center, Oklahoma City, Oklahoma 73104, USA; 4 Harold Hamm Diabetes Center, University of Oklahoma Health Sciences Center, Oklahoma City, Oklahoma, 73104, USA

## Abstract

Imatinib (Gleevec), a non-receptor tyrosine kinase inhibitor (nRTKI), is one of the most successful anti-neoplastic drugs in clinical use. However, imatinib-resistant mutations are increasingly prevalent in patient tissues and driving development of novel imatinib analogs. We present a detailed study of the conformational dynamics, in the presence and absence of bound imatinib, for full-length human c-Src using hydrogen-deuterium exchange and mass spectrometry. Our results demonstrate that imatinib binding to the kinase domain effects dynamics of proline-rich or phosphorylated peptide ligand binding sites in distal c-Src SH3 and SH2 domains. These dynamic changes in functional regulatory sites, distal to the imatinib binding pocket, show similarities to structural transitions involved in kinase activation. These data also identify imatinib-sensitive, and imatinib-resistant, mutation sites. Thus, the current study identifies novel c-Src allosteric sites associated with imatinib binding and kinase activation and provide a framework for follow-on development of TKI binding modulators.

Cellular Src kinase (c-Src, Fig. 1) is a non-receptor tyrosine kinase (nRTK) involved in modulating signaling cascade activity – including the MAPK, EGFR, and GPCR-mediated pathways^1^,^2^,^3^. Aberrant regulation of c-Src activity is associated with cancer, diabetes, and chronic inflammation in humans. Thus, development of c-Src specific inhibitors is of paramount clinical importance^1^,^2^,^3^. A majority of tyrosine kinase inhibitors (TKIs) used in clinical practice were developed to target the nRTK ATP binding pocket and can be divided into two classes (Type I and Type II) based upon conformation of the conserved DFG-motif in crystal structures of TKI-nRTK complexes^4^,^5^. Type I inhibitors consist of ATP-competitive inhibitors that bind to kinase conformers with the DFG motif (residue 404–406 in c-Src) side chains oriented towards the ATP binding pocket (DFG-in) while type II inhibitors, including imatinib (Gleevec), bind to conformers with the DFG motif side chains oriented away from the ATP binding pocket (DFG-out). Orientation of the DFG motif side chains plays a critical role in nRTK drug binding – as evidenced by a comparison of c-Src to a closely related nRTK named c-Abl. Imatinib binds c-Src (DFG-in predominates) with a lower affinity relative to c-Abl (DFG-out predominates in native state) which is attributed to differences in their DFG motif side chain orientations^5^,^6^,^7^. Additional differences are observed when comparing imatinib-bound c-Abl and c-Src structures: while both c-Src and c-Abl form hydrogen bonds with the backbone of Met341 (Met337 in c-Abl) with imatinib (Fig. 1c), c-Abl forms additional hydrogen bonding interactions with imatinib via the side chain of gate keeper Thr334 (c-Abl numbering) and the backbone of Asp400 (c-Abl numbering) of the DFG motif 8,9. Additional structural regions must prime the chemical environment of the ATP binding pocket to optimize imatinib interactions since replacement of the c-Src ATP binding pocket residues with corresponding c-Abl residues by mutagenesis does not result in the same imatinib binding affinity displayed by c-Abl^6^. Role of the DFG motif in determining imatinib binding specificity and affinity has been studied primarily by X-ray crystallography, NMR, and MD simulations using isolated kinase domains^7^,^10^,^11^,^12^,^13^. These studies provided additional information about important structural elements for imatinib binding in atomic detail, including: exposure of an activation-loop (A-loop) containing Tyr416 auto-phosphorylation site, rotation of helix C, and re-organization of the interface between kinase domain N- and C-lobes that result in stabilization of a hydrophobic spine composed of Leu325, Met314 in helix C, DFG, and the HRD motif (Fig. 1c–e). These structural changes stabilize a DFG-out conformer in a native state ensemble, suggesting conformational selection of imatinib binding^7^. Further effort to study c-Src native state dynamics has been hampered by the absence of DFG motif NMR signals and the extremely slow transition to a DFG-out conformation that is not accessible to conventional MD simulation timescales^7^,^14^. A recent study proposed an additional mechanism underlying imatinib affinity differences where fast equilibration between DFG-in and DFG-out is followed by slow accumulation of kinase-imatinib complexes (E^*^-I) via an induced fit mechanism^11^, although structural features of this E*-I complex are not yet known. A critical missing piece to solve the molecular mystery of imatinib binding requires information about protein dynamics that links snapshots of crystal structures in different states. The emergence of imatinib resistant mutations highlights the importance of such studies for developing new classes of specific TKIs^15^,^16^,^17^. This may require identification of potential drug target sites distal to the highly conserved kinase domain ATP binding pocket.

We expressed and purified full-length human c-Src containing the SH3, SH2, and kinase domains (residue 83-533 based on the numbering of PDBID:1Y57) to probe c-Src dynamics in the absence, and presence, of imatinib using hydrogen-deuterium exchange and mass spectrometry (HDX-MS). HDX-MS results demonstrate that the ATP binding pocket and hydrophobic spine (excluding the binding cradle containing Met341) are structurally rigid in the absence of imatinib. Imatinib binding results in similar structural dynamics to structural changes involved in the kinase activation steps. Of note, the effects of imatinib binding are not confined within the kinase domain itself. Instead, HDX-MS unambiguously demonstrates propagation of structural dynamics from the imatinib binding site to distal functional regulatory sites in the SH3 and SH2 domains. Analysis of clinically-identified imatinib resistant mutation sites, together with our HDX-MS results, provides intriguing insight into the relationship between allostery and evolution of drug resistant mutations.

## Results

### HDX-MS indicates a stable hydrophobic spine in unphosphorylated c-Src

**Figure 1:**
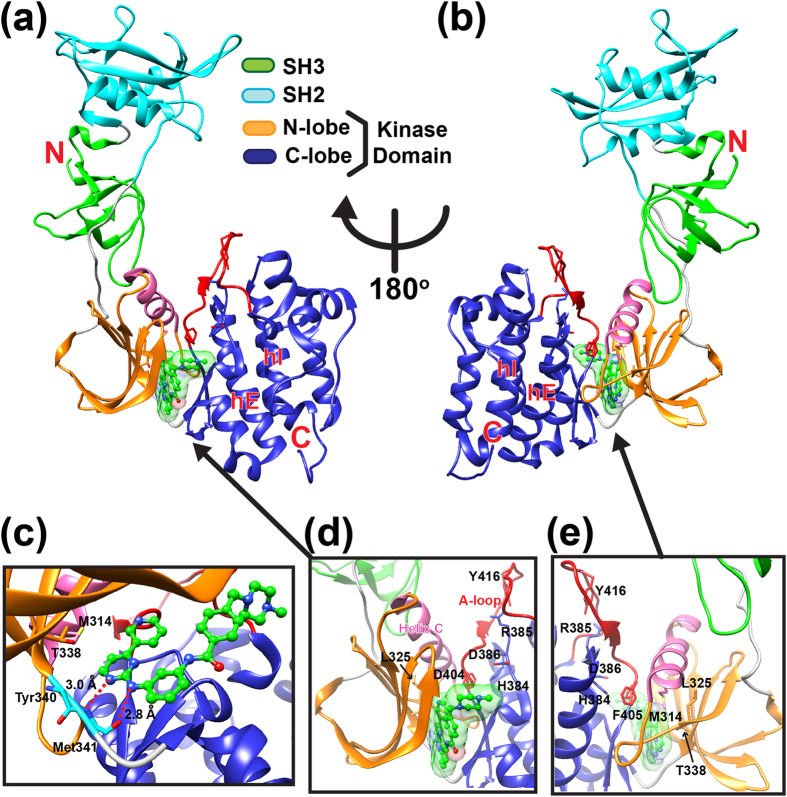
Crystal structure of the unphosphorylated human c-Src bound to imatinib. **(a**,**b)** Crystal structure of human c-Src previously solved by X-ray crystallography (PDB ID:1Y57)^9^. Each domain is shown in different colors with the N- and the C-terminus indicated. Helix C and activation loop (A-loop) are shown in pink and red, respectively. The bound imatinib is shown in green stick with surface representation. Helix E (hE) and helix I (hI) are labeled. In (**b**) the c-Src molecule in (**a**) is rotated 180° about y-axis. **(c)** A close-up view of imatinib binding site. The backbone of Tyr340 and Met341 is shown in cyan sticks, and imatinib is shown in green ball-and-sticks. The hydrogen bond distance between the backbone of Met341 and imatinib is indicated. Gatekeeper Thr338 and Met314 in helix C are also indicated. **(d**,**e)** A close view of the hydrophobic spine region with the HRD and DFG motifs indicated.

**Figure 2:**
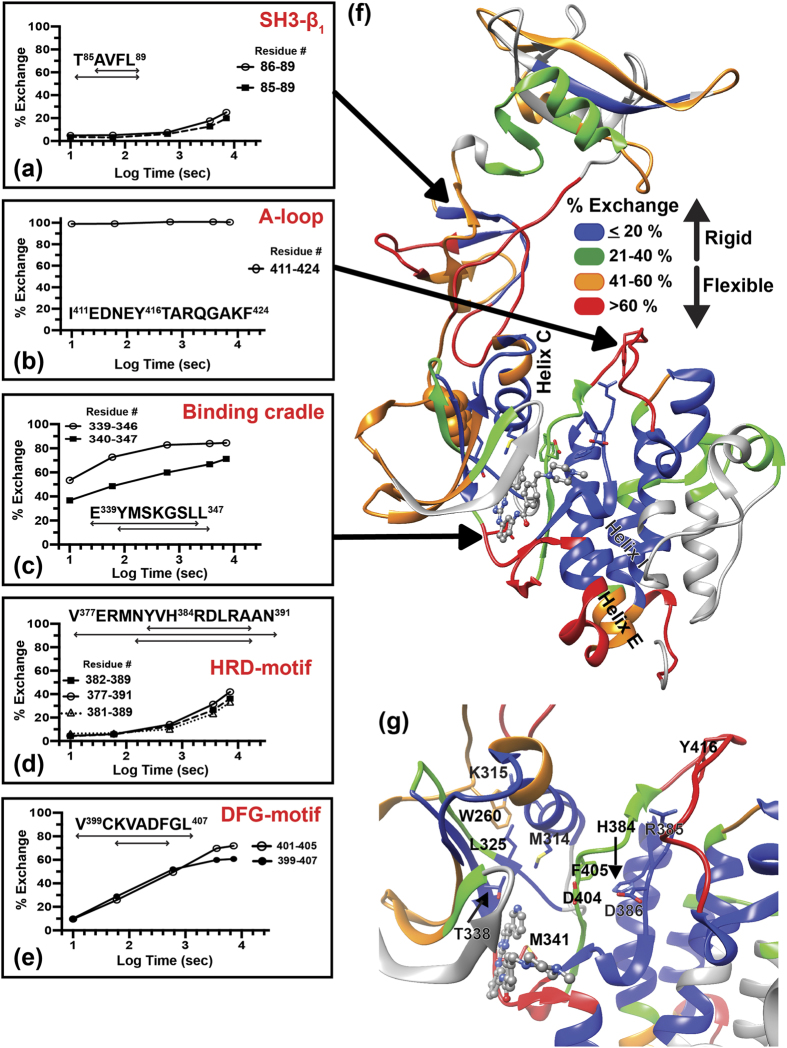
HDX-MS of unphosphorylated human c-Src without imatinib. **(a**–**e)** The percent deuterium exchange as a function of deuterium labeling time in logarithmic scale for indicated peptides relative to the percent exchange of the corresponding peptide derived from unfolded and fully deuterated c-Src as described in *Methods*. The amino acid sequence and residue number for each peptide are indicated in figures. Double-headed arrows under amino acid sequence indicate the length of peptide. **(f)** The percent exchange at 1 min labeling time mapped onto the c-Src crystal structure. Different regions are colored according to the percent exchange of each peptide. Grey regions indicate regions with standard deviation of greater than ±0.1 Da from triplicate experiments; therefore, excluded from analysis. Imatinib binding site is indicated in grey transparent surface representation. **(g)** Close-up view of hydrophobic spine region with the percent exchange at 1 min mapped onto the structure. Functionally important residues are labeled.

To investigate the effect of imatinib binding on the dynamics of human c-Src, we expressed and purified c-Src in the unphosphorylated form as described previously^18^. Ample experimental evidence demonstrates the importance of inter-domain communication in regulating c-Src activation^19^,^20^,^21^,^22^. If allosteric communication exists between domains then structural perturbations in the ATP binding pocket following imatinib binding should influence dynamics of distal SH3 and SH2 domains. To test this hypothesis, we first monitored dynamics of unphosphorylated c-Src in the absence of imatinib using HDX-MS. Briefly, backbone amide hydrogen atoms undergo deuterium exchange when they are solvent-exposed while amide hydrogen atoms engaged in hydrogen bonds or buried in hydrophobic environment are protected from the exchange^23^. In order for these hydrogen bonded or buried hydrogen atoms to exchange with deuterium, breaking hydrogen bonds or partial unfolding is necessary^23^. By monitoring the number of deuterium atoms incorporated in each peptic fragment as a function of deuterium labeling time, structural fluctuations in native state ensemble are probed in a region-specific manner (Fig. 2a–e). Purified human c-Src was incubated in D_2_O, and the labeling was quenched at different time points followed by pepsin digestion and mass spectrometry analysis. To aid visualization of flexible and rigid regions in the unphosphorylated c-Src structure, the percent deuterium exchange in different regions is assigned different colors and mapped onto the c-Src crystal structure (Fig. 2f,g). Distributions of flexible and rigid regions in the c-Src structure are evident. For example, HDX-MS shows that local structural rigidity of a β-strand in SH3 domain results in low deuterium uptake (Fig. 2a) while flexibility of the loop containing Tyr416 activating autophosphorylaiton site (A-loop) leads to immediate exchange with deuterium in 10 sec (Fig. 2b). In the unphosphorylated c-Src-imatinib complex crystal structure, imatinib fills in the ATP binding pocket and is cradled on a loop by making two hydrogen bonds with the backbone of Met341 (Fig. 1c)^9^. Structural flexibility in this binding cradle (Fig. 2c,f) suggests importance of this region in aiding imatinib entry into the ATP binding pocket. Comparison of the percent exchange of two peptides in the binding cradle, 336–340 and 336–341, shows that incorporation of Tyr340 accounts for a nearly 30% increase in the percent deuterium exchange at 1 min labeling time (Supplemental Fig. 1), suggesting that the flexible binding cradle segment includes residues 341–346 as well as Tyr340. Inclusion of Tyr340 as a flexible binding cradle residue is also corroborated by high deuterium percent exchange (65%) in peptide 339–346 (Fig. 2c). Although the binding cradle is extremely flexible, a majority of both lobes are structurally rigid. Indeed, a cluster of rigid regions, consisting of α-helices, is seen in the kinase domain C-lobe (Fig. 2d–g). Regions containing hydrophobic spine residues are also protected from deuterium exchange (Fig. 2d–g). These include Leu325, Met314 in helix C, His384 and Asp385 of the HRD motif (Fig. 2d), and Asp404 and Phe405 of the DFG motif (Fig. 2e–g). Gatekeeper Thr338, a key residue in stabilizing a DFG-out conformation, is also located in a structurally rigid region (Fig. 2g). These hydrophobic spine residues run across the N- and the C-lobes, and together with helix E (Fig. 2f), these regions form an interface between the kinase domain N- and the C-lobes that is not readily accessible to deuterium (Fig. 2f–g). In particular, structural rigidity of helix E in the C-lobe must be important in stabilizing the interface as imatinib resistant mutation sites including Met366, Met374, and Glu378 are localized in this helix^16^. These residues are situated adjacent to another imatinib resistant mutation site, F509 in helix I that packs against helix E (Fig. 2f). These observations imply that the loss of structural packing between helices E and I could be associated with development of imatinib resistance. Although a majority of kinase domains contain clusters of solvent-inaccessible hydrophobic core residues, structurally rigid regions in both the SH3 and SH2 domains are largely absent except short N-terminal β-sheets and central β-strand in the SH3 and SH2 domains, respectively (Fig. 2a,f). Structural fluctuations within these domains as well as in a loop connecting these domains suggests considerable domain motions relative to the kinase domain that may be significant in recognizing a wide range of binding partners. A central loop connecting the SH2 and kinase domains is also flexible, particularly in the N-terminal half of the loop. The C-terminal side of the connecting loop contains highly conserved Trp260 that engages in a cation-π interaction with opposing Lys315 in helix C (Fig. 2g). The protection from the deuterium exchange in Lys315-containing regions implies importance of the Trp260-Lys315 interaction in anchoring the kinase domain to the C-terminal half of the loop (Fig. 2f,g). Because a W260A mutation results in constitutive activation of c-Src^21^, disruption of this anchoring appears to be important in regulating c-Src activity.

### Imatinib binding disassembles the C-Src hydrophobic spine

**Figure 3:**
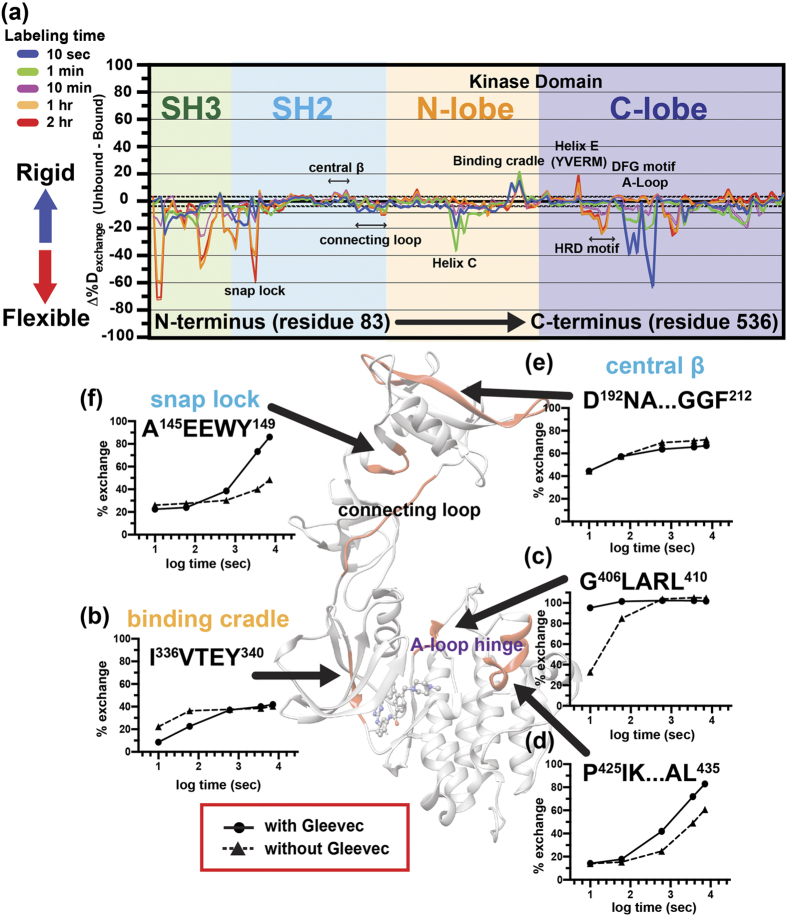
Dynamics of the human c-Src-imatinib complex. **(a)** Difference plot for imatinib binding. The location of each domain is colored. Functionally important regions are indicated with double-headed arrows. The difference in the percent exchange between imatinib unbound and bound c-Src (Δ%D_exchange_) at different deuterium labeling time points for all analyzed peptides is shown in different colored lines. Black-dotted and thick horizontal lines indicate 98% confidence limit for individual time points (colored lines, CL Δ%D_exchange_ ± 3.52%). **(b**–**f)** The percent deuterium exchange of indicated regions as a function of deuterium labeling time on a logarithmic scale in the absence (black triangle) or the presence (black circle) of imatinib. The amino acid sequence of peptides is shown in each figure. The location of each peptide in c-Src structure (center) is colored in red. Location of the SH2-kinase domain connecting loop is also indicated in the structure.

Imatinib binding to c-Src was first monitored by changes in the intrinsic tryptophan fluorescence upon imatinib titration (Supplemental Fig. 2a–c). The obtained apparent K_D_ (42 μM) is in agreement with a previously reported K_D_ obtained with the isolated kinase domain^6^. For HDX-MS experiments, c-Src was pre-incubated in imatinib-H_2_O buffer for 30 min before the addition of imatinib-D_2_O buffer. This continuous D_2_O labeling technique was employed to probe the structural dynamics of a c-Src-imatinib complex. HDX-MS results unambiguously indicate changes in the c-Src dynamics upon imatinib binding (Fig. 3). Such changes are summarized in the difference plot, showing the difference in the percent deuterium exchange between imatinib-unbound and imatinib-bound forms, ∆%D_exchange_ (Fig. 3a). Structural rigidity in the binding cradle is evident as shown in the percent exchange of the Y340-containing peptide (Fig. 3a,b): at 10 sec labeling time, Tyr340-containing peptide, I^336^VTEY^340^, shows a significant reduction in the percent exchange upon imatinib binding compared to that of the same peptide in the unbound form (Fig. 3b). Similarly, when the same peptide is compared at 10 min labeling time, the percent exchange of both imatinib-bound and -unbound forms become comparable, assigning Tyr340 as a residue undergoing a rigid to flexible structural transition in the presence of imatinib (Fig. 3b). Imatinib binding also impacts another key hinge region that modulates the A-loop dynamics: the N-terminal side of A-loop hinge region, G^406^LARL^410^, has a significant increase in deuterium uptake at short labeling time of 10 sec and 1 min (Fig. 3c) while C-terminal side of the A-loop hinge, spanning residue 425 to 435, shows no distinct change in the percent deuterium exchange at those labeling time points (Fig. 3d). The elevated deuterium uptake in G406-L410 coincides with increased DFG motif flexibility (Fig. 3a), indicating increased solvent accessibility in the hydrophobic spine region upon imatinib binding. Indeed, increased structural fluctuations in both helix C and the HRD motif (Fig. 3a) suggest disassembly of the hydrophobic spine with concomitant loosening of the interface between the N- and C-lobes.

Our results also demonstrate that despite the lack of direct contacts between hydrophobic spine residues and imatinib, dynamic changes in the binding cradle are propagated across the kinase domain. For example, changes in structural fluctuations of the A-loop hinge region (Fig. 3c,d) suggest allosteric communication between the A-loop and binding cradle in the presence of imatinib. If such communication exists, imatinib binding should be affected by Tyr416 phosphorylation. To test this idea, c-Src was subjected to a kinase reaction, and the reacted protein was analyzed by western blot using specific pTyr416 or non-phosphorylated c-Src antibody. Only a band corresponding to pTyr416 c-Src was detected, indicating homogeneously phosphorylated c-Src at Tyr416 (Supplemental Fig. 3a,b). The binding was then monitored by changes in the intrinsic tryptophan fluorescence of c-Src upon imatinib titration (Supplemental Fig. 3c,d). Autophosphorylation at Tyr416 results in decreased imatinib binding affinity with the apparent K_D_ of 63 μM, supporting the idea of allosteric communication between the A-loop and binding cradle.

The effect of imatinib binding on c-Src dynamics is not confined within the kinase domain as HDX-MS clearly indicates additional changes in SH3 and SH2 domain dynamics (Fig. 3a). Specifically, regions involved in functional regulation show changes upon imatinib binding. For instance, imatinib binding results in reduced deuterium uptake in a phosphopeptide ligand binding site including the central SH2 domain β-strand (residue 192–212, Fig. 3a,e). On the other hand, the N-terminal half of the SH2-kinase connecting loop spanning residues 246 to 255 show increased structural fluctuations (Fig. 3a). This region forms a polyproline type II helix (PPII) that interacts with the SH3 domain to facilitate compaction of the entire molecule in the inactive pTyr527 state^24^. Increased flexibility is also found in the SH3-SH2 connecting loop region, referred to as a “snap lock”, that clamps both SH3 and SH2 domains together in response to inactivating Tyr527 phosphorylation (Fig. 3a,f)^22^. The increased snap lock fluctuations coincide with structural unraveling of the entire SH3 domain (Fig. 3a), substantiating a previous proposal that snap lock couples SH3 and SH2 domain motion. These observations further suggest allosteric communication between the ATP binding pocket and distant functional regulatory sites.

### Dynamics of imatinib resistant mutation sites suggest inter-lobe allostery

**Figure 4:**
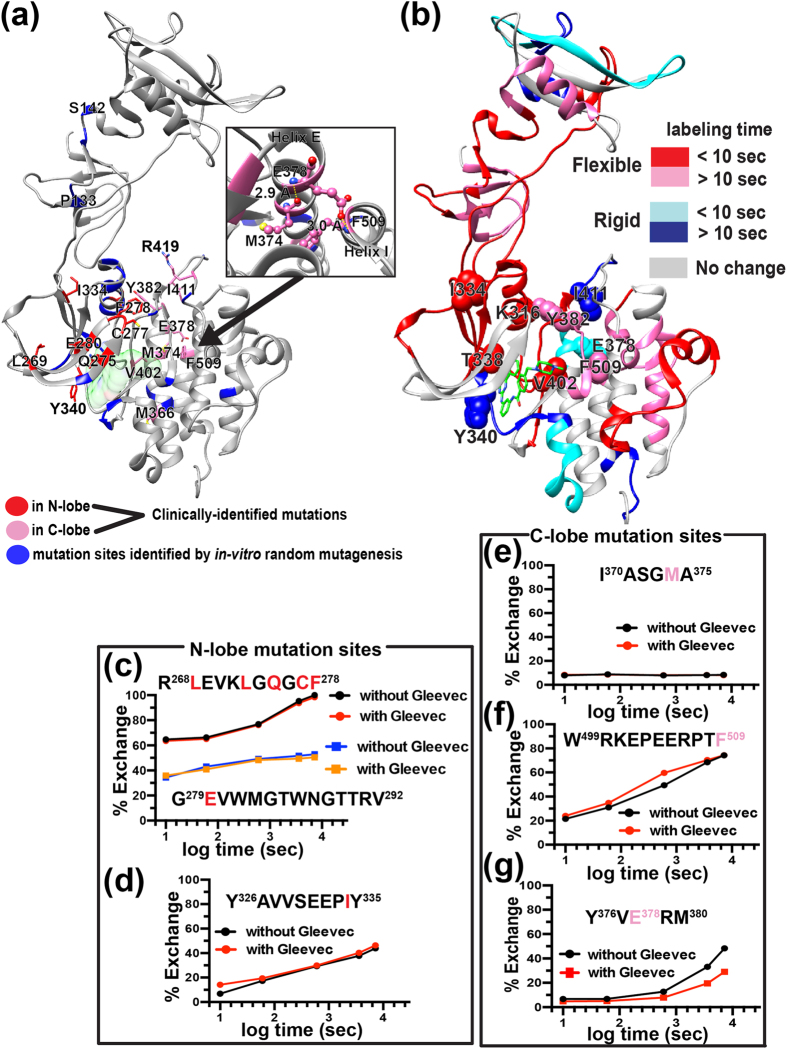
Imatinib binding sensitive and insensitive resistant mutation sites. **(a**,**b)** Imatinib resistant mutation sites identified in previous studies^16^,^25^ are mapped onto the c-Src crystal structure. The resistant mutation sites identified in Ba/F3 cells or clinical samples are highlighted in blue or red/pink, respectively. Only conserved residue mutation sites are highlighted. **(b)** A structure map of Δ%D_exchange_. Grey regions indicate regions with no change in Δ%D_exchange_. Red and pink regions indicate increased deuterium uptake (Δ%D_exchange_) above a statistically significant limit (+3.52%) at 10 sec or longer than 10 sec (pink) labeling time points. Similarly, blue and cyan regions show decreased deuterium uptake above statistically significant limit (−3.52%) at 10 sec (cyan) or longer than 10 sec labeling time (blue). Imatinib resistant mutation sites are shown in spheres with residue numbers labeled. **(c**,**d)** The percent deuterium exchange plot of indicated peptides in N-lobe with (red line) or without (black line) imatinib. Imatinib resistant mutation sites in N-lobe are highlighted in red in the amino acid sequence of peptides. **(e**–**g)** The percent deuterium exchange plot of mutation site-containing peptides in C-lobe with (red line) or without (black line) imatinib. Resistant mutation sites are highlighted in pink in the amino acid sequence.

Previous studies identified imatinib resistant mutation sites in c-Abl by *in vitro* random mutagenesis and by Sanger sequencing of clinical samples^17^,^25^,^26^,^27^,^28^,^29^. The corresponding single mutation sites in c-Src were mapped onto a c-Src structure (Fig. 4a). To look into possible mechanisms of imatinib resistance, both amino acid sequence conservation and ∆%D_exchange_ are mapped onto the same c-Src structure (Fig. 4b, Supplemental Fig. 4a,b). N-lobe contains mutation sites that are associated with severe imatinib resistance. For example, a point mutation at conserved F278 or E280 (Y253 or E255 in c-Abl numbering, respectively) in N-lobe (Fig. 4a) increases the IC_50_ to a comparable level to that of the well-known gatekeeper mutant T338I, with IC_50_ increases ranging from 29 to 45 fold compared to that of wild-type^16^,^26^. While F278 or E280-containing peptide shows no apparent change in the deuterium uptake upon imatinib binding (Fig. 4c), the deuterium uptake of conserved gatekeeper T338-containing peptide increases slowly over two hours (Fig. 3b). This implies a lack of stable hydrogen bonding interaction between imatinib and the gatekeeper T338 residue. Instead, HDX-MS results suggest that the binding cradle containing Tyr340 aids the interaction between c-Src and imatinib (Figs 3b and 4c). The importance of Tyr340 in imatinib binding is also supported by findings where a point mutation at Tyr340 results in a 3–4 fold IC_50_ increase^16^,^26^. Overall, except F278 and E280-containing β-strands, the majority of N-lobe experiences increased structural flexibility upon imatinib binding (Fig. 4c). This also includes another conserved residue, I334 (Supplemental Fig. 4a), that shows a modest increase in deuterium uptake (10%) at the 10 sec labeling time point upon imatinib binding (Fig. 4c,d), and a point mutation at I334 increases the IC_50_ only two-fold^16^,^26^. These observations indicate that for N-lobe resistant mutation sites, neither the amino acid sequence conservation nor ∆%D_exchange_ correlates with severity of imatinib resistance.

In contrast, such a correlation is seen in C-lobe mutation sites. For example, deuterium uptake of a Y382 (Fig. 3e) or V402 (Fig. 3c) containing peptide shows substantial increase upon imatinib binding, and a point mutation at either residue increases IC_50_ 11 to 16 fold^16^,^26^. On the other hand, M374- or F509-containing peptide shows no or modest change in ∆%D_exchange_ (10%), respectively, (Fig. 4e,f) with a point mutation at either residue increasing IC_50_ only 3–5 fold^16^,^26^. In the imatinib-bound c-Src crystal structure, M374 and F509 form a network of hydrogen bonds with another imatinib resistant mutation site, E378 (Fig. 4a, inset)^9^. A point mutation at E378 in helix E is one of the most frequently observed imatinib resistant mutations in patients that produces a significant IC_50_ increase (ten-fold)^16^. The structural rigidification of helix E containing Y^376^VERM^380^ (Fig. 4g) indicates that a mutation at E378 would disrupt this hydrogen bonding network with concomitant loss of helical packing between helices E and I. This could be an underlying cause of imatinib resistance resulting from a E378 mutation: changes in the structural dynamics of helices E and I as a result of the loss of the helical packing is likely to be propagated to the binding cradle via the inter-lobe interface, suggesting that a disruption of such inter-lobe allostery could also be associated with imatinib resistance.

## Discussion

Since the mechanism of imatinib action involves stabilization of the unphosphorylated form to block kinase activation^5^, it is essential to study changes in the structural dynamics of c-Src in the absence and presence of imatinib. Using HDX-MS, we probed the structural dynamics of a canonical nRTK c-Src to identify allosteric sites that may modulate the interaction between imatinib and c-Src. Previous studies suggest the DFG motif conformation as a critical determinant in imatinib binding specificity and affinity^4^,^5^,^7^. HDX-MS results show that the DFG-motif is located in a structurally rigid region in the absence of imatinib, (Fig. 2e–g) implying inherent difficulty for c-Src to undergo interconversion between DFG-in and DFG-out conformations in its native state ensemble. This finding is substantiated by a previous computational study where the stability difference for DFG-in and DFG-out conformations (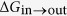) between c-Abl and c-Src were found to be 5.4 kcal/mol and 1.4 kcal/mol, respectively^7^. This previous result, together with our current findings, provide insight into the kinetic and thermodynamic basis of conformational switching between DFG-in and DFG-out that regulates imatinib binding.

HDX-MS results demonstrate that imatinib binding influences the structural dynamics of the hydrophobic spine. For example, drug binding produces concerted structural changes in the HRD motif and C-terminal A-loop hinge, spanning residues 425 to 435, as implied by similar deuterium uptake kinetics of both regions occurring over 10 min to two hours (Fig. 3a,d). Further analysis of ∆%D_exchange_ suggests that the hydrophobic spine is already assembled in the unphosphorylated form while imatinib causes its disassembly (Fig. 3a). This observation is a striking contrast to a previous proposal where the hydrophobic spine is assembled only in the active Tyr416 phosphorylated (pTyr416) form^10^. Our observation of concerted dynamics in the hydrophobic spine region is consistent with a previous MD simulation study where hydrophobic spine destabilization leads to increased mobility of helix C that facilitates ATP binding^12^. The same previous study also suggested that E310 in helix C, together with R409 in the N-terminal A-loop hinge, forms a crucial interaction in stabilizing the partially activated intermediate state that aids the inactive to active transition^12^. Increased structural fluctuations in regions containing either of these two residues (Fig. 3a,c) suggest an inability of c-Src to form partially activated intermediates in the presence of imatinib; therefore, it is unable to make a full transition to the active form. Yet, imatinib-bound c-Src appears to undergo structural changes required for c-Src activation. Such structural changes include increased flexibility in the entire helix C and DFG motif (Fig. 3a), as well as increased HRD motif solvent accessibility (Fig. 3a)^12^,^30^, suggesting loosening of the kinase domain interface formed by the N- and the C-lobes. These observations imply that although imatinib is a type II non-ATP analog TKI^5^, imatinib binding induces structural transitions similar to changes involved in ATP binding, but imatinib-bound c-Src appears to be trapped in a pre-activated state. This pre-activated structural state may be similar to the E-I* conformer that was detectable by ^1^H-^15^N HSQC NMR only when the data acquisition was extended to 16 hours^11^.

Regions distal to the kinase domain also undergo similar structural transitions involved in activation upon imatinib binding. Such regions consist of functional regulatory sites including the central β-strand in the SH2 domain and snap lock in SH3-SH2 connecting loop that show increased structural fluctuations (Fig. 3a,f). Moreover, deuterium uptake of the SH3 domain indicates structural unraveling of the entire SH3 domain in the presence of imatinib (Fig. 3a), suggesting strong allosteric coupling between the ATP binding pocket and SH3 domain conformation. This is also corroborated by a previous study where an SH3 domain destabilizing mutation results in activation, and subsequent loss of proline-rich ligand binding activity^20^,^31^,^32^,^33^. These findings have an important implication in devising a new kinase inhibition strategy. For example, a reagent that modulates SH3 or SH2 domain dynamics could be introduced together with currently available orthosteric TKIs to improve its binding affinity and specificity. Development of such reagents (allosteric modifiers) expands the repertoire of drug design strategy. Because this type of combined drug therapy relies more on the concentration of modifier than that of orthosteric TKI, this idea may also help mitigate development of drug resistance mutations.

Although targeted drug therapies such as imatinib revolutionized treatment protocols for chronic myeloid leukemia (CML), the occurrence of drug resistance and relapse are still prevalent^16^,^17^,^27^. We show distinct dynamics of both N- and C-lobes in response to imatinib binding: most drug resistant mutation sites in the N-lobe experience increased structural fluctuations while C-lobe helix E, made of the lobe interface, undergoes structural rigidification (Fig. 4b). Such opposing dynamics at the lobe interface suggest the loss of concerted motion between the two lobes that may be important in modulating A-loop dynamics to regulate activation. Because A-loop bridges N- and C-lobes, its dynamics must be dependent on allosteric communication between the two lobes. It then follows that imatinib binding may disrupt such inter-lobe allosteric communication. Furthermore, analysis of clinically identified resistant mutation sites provide us intriguing insight into a relationship between allostery and evolution of drug resistant mutations. One such example is seen in helix E that contains abundant compound mutation sites - mutations that are acquired subsequent to the emergence of “seeding” mutation sites including M374 and E378 in helix E, and gate keeper T338 (Supplemental Fig. 5)^29^. Interestingly, compound mutations tend to appear in the opposite lobe to the seeding mutation sites. For example, clinical samples carrying a mutation at M374 often harbor more than one compound mutation in the same molecule including V323, R359, I370, Y463, V467, or E486^29^. Another seeding mutation in helix E, E378 (E355 in c-Abl), results in the emergence of compound mutations mainly in the N-lobe^29^. Furthermore, while the gatekeeper T338I mutation has been extensively studied, clinical isolates carrying T338I are associated with the appearance of compound mutations at T290, T354, A375, or Y382^29^. The introduction of additional mutations is expected to influence the dynamics and function of c-Src and imatinib binding through inter-lobe allostery. Further studies focused on the biochemical/biophysical properties of c-Src carrying compound mutations may provide additional strategies for developing new classes of kinase inhibitors.

## Methods

### Expression and purification of unphosphorylated human c-Src (residue 83-536) and preparation of pTyr419 c-Src

Human c-Src was cloned, expressed in *E.coli*, and purified as described in our previous study^18^. Briefly, human c-Src was expressed in Rosetta(DE3)pLysS cells (Novagen) followed by cell lysis by sonication (Sonics Vibra Cell VCX500 with 13 mm probe 630-0220) at 50% amplitude (30 sec on, 30 min off; 10 cycles) on ice. Total cell lysate was centrifuged at 26,000 × g, and human c-Src was purified by two chromatographic steps, cobalt column followed by Hitrap-Q-anion exchange column (GE Healthcare) as described previously. Size exclusion chromatography was carried out using a Superdex 200 10/300 GL column (GE Healthcare) for buffer exchange into appropriate buffer for different experiments in this study.

### Western blot analysis

The purified c-Src (2 μM) in 50 mM HEPES (pH 7.4), 100 mM NaCl, and 1 mM TCEP was incubated with 5 mM MgCl_2_ and 5 mM ATP at 37 °C for 24 hours to carry out the autophosphorylation reaction at Tyr416. The reaction samples were centrifuged, and the supernatant samples (500 ng) were run on 10% SDS-PAGE at 200 V for one hour followed by a transfer onto PVDF transfer membrane (Thermo Scientific) for 30 min at 0.3 milliamps using Trans-Blot SD semi-dry transfer cell (Bio-Rad). The blot was blocked with Tris-buffered saline/Tween-20 buffer containing 5% non-fat dry milk powder at 4 °C overnight prior to the primary antibody incubation with either anti-pTyr416 or anti-non-phospho-Tyr416 antibody (catalog# 6943S or 2102P from Cell Signaling Technology) for one hour at room temperature. The membrane was washed with Tris-buffered saline/Tween-20 for 10 min, and the wash was repeated three times. The membrane was then incubated with HRP-conjugated secondary anti-rabbit or -mouse IgG antibody (Cell Signaling Technology) for one hour at room temperature. Subsequently, the membrane was washed with Tris-buffered saline/Tween-20 buffer for 10 min with three repeated washes. The absence or the presence of phosphorylation at Tyr416 was visualized on X-ray films with the exposure time of three seconds using chemiluminescent substrate (Thermo Scientific).

### Imatinib binding monitored by fluorescence spectroscopy

Intrinsic tryptophan fluorescence of purified unphospho- or pTyr416 phosphorylated c-Src in 20 mM sodium phosphate (pH 7.4) and 100 mM NaCl at 25 °C was monitored using an ISS photon counting spectrofluorometer. Imatinib (Santa Cruze Biotech) was freshly prepared in water at 10 mg/mL stock concentration just before each experiment as described previously^34^ except the lyophilization step was omitted. The protein (0.1 μM) was incubated with different concentrations of imatinib for at least 10 min at 25 °C before measurements. The excitation wavelength was set to 295 nm, and the emission wavelength from 310 to 420 nm was monitored at 25 °C. Each sample was scanned three times, and each experiment was repeated in triplicate. Differences of tryptophan fluorescence intensity (∆F) at 340 nm in the absence (F_0_) or the presence of different concentration of imatinib (F) was normalized to the maximum tryptophan fluorescence intensity observed at 200 μM imatinib (F_max_) to obtain the fraction of imatinib-bound c-Src as the following:


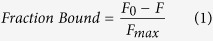


The fraction of imatinib-bound c-Src was plotted against the concentration of imatinib, and the data were fitted to a Langmuir isotherm 1:1 binding model to obtain apparent K_D_ using Prism 6 (GraphPad software, Inc.).

### Hydrogen-Deuterium Exchange and Mass spectrometry

Preparation of deuterium-labeled c-Src and HDX-MS experiments were carried out as described previously^35^. Purified unphosphorylated c-Src (100 pmol) in 20 mM sodium phosphate (pH 7.4) and 100 mM NaCl-H_2_O buffer was diluted with D_2_O buffer (Cambridge Isotope Laboratories) 15 fold to label amide hydrogen atoms. The labeling reaction was carried out at 25 °C for different time periods (10 section, 1 min, 10 min, 1 hr or 2 hrs), an the reaction was quenched by addition of cold 100 mM sodium phosphate buffer at pH 2.4 containing 2 M guanidine hydrochloride. For imatinib-containing samples, unphosphorylated c-Src (100 pmol) was incubated with freshly prepared imatinib at the final concentration of 300 μM for 30 min at 25 °C prior to deuterium labeling. The D_2_O buffer contained 20 mM sodium phosphate (pD 7.4), 100 mM NaCl, 300 μM imatinib, and the labeled sample was quenched by addition of cold 100 mM sodium phosphate buffer (pH 2.4) containing 2 M guanidine hydrochloride. The quenched sample was immediately injected onto a Waters HDX nanoAcquity UPLC (Waters, Milford, MA) with in-line pepsin digestion (Poroszyme immobilized pepsin cartridge from Applied Biosystems). Peptic fragments were trapped on an Acquity UPLC BEH C18 peptide trap and separated on an Acquity UPLC BEH C18 column. A 7 min, 5 to 35% acetonitrile (0.1% Formic acid) gradient was used to elute peptides directly into a Waters Synapt G2 mass spectrometer (Waters, Milford, MA). MS^E^ data were acquired with a 20 to 30 V ramp trap CE for high energy acquisition of product ions as well as continuous lock mass (Leu-Enk) for mass accuracy correction. Peptide identification and protein sequence coverage maps were obtained from undeuterated controls. Peptides were identified using the ProteinLynx Global Server 2.5.1 (PLGS) from Waters. Fully deuterated controls were performed by incubating in the presence of 6M Guanidine DCl for two hrs prior to quenching. All deuterium incubation time points and controls were repeated three times. Data and statistical analysis were carried out as described previously^35^,^36^. Briefly, the deuterium uptake by the identified peptic fragments through increasing deuteration time and for the fully deuterated control was determined using Water’s DynamX 2.0 software (Waters). The normalized percentage of deuterium uptake at incubation time t (%D_t_) at an incubation time *t* for a given peptide was calculated as follows:


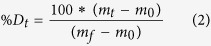


With *m*_*t*_ being the centroid mass at incubation time *t*, *m*_*0*_ the centroid mass of the undeuterated control and m_f_ the centroid mass of the fully deuterated control. For any given peptide, the percent deuteration difference plots Δ%D_exchange_ (Unbound-Bound) presented in Fig. 3a were generated by subtracting the corresponding percent deuteration at incubation time t calculated for the imatinib bound from the unbound as the following:


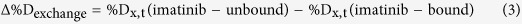


where X is each peptide, and t is D_2_O incubation time.

Confidence intervals for the Δ%D_exchange_ were determined using the method outlined by Houde *et al.*^36^ adjusted to percent deuteration using the fully deuterated controls. Accordingly, 98% confidence interval of +/−3.52%D for any single time point and ++/−7.87%D for the sum of all five incubation time points were determined.

## Additional Information

**How to cite this article**: Tsutsui, Y. *et al.* Imatinib binding to human c-Src is coupled to inter-domain allostery and suggests a novel kinase inhibition strategy. *Sci. Rep.* **6**, 30832; doi: 10.1038/srep30832 (2016).

## Supplementary Material

Supplementary Information
