# Supplementary material for: Imatinib binding to human c-Src is coupled to inter-domain allostery and suggests a novel kinase inhibition strategy
Source: Sci Rep. 2016 Aug 2;6:30832. doi: 10.1038/srep30832 (PMC4969603; doi:10.1038/srep30832)
Supplement: Supplementary Information [file srep30832-s1.pdf]

SUPPLEMENTARY FIGURES

Imatinib binding to human c-Src is coupled to inter-domain allostery and suggests a novel kinase inhibition strategy

Yuko Tsutsui[1], Daniel Deredge[2], Patrick L. Wintrode[2], and Franklin A. Hays[1,3,4,*]

[1]Department of Biochemistry and Molecular Biology, University of Oklahoma Health Sciences Center, Oklahoma City, Oklahoma 73104, USA.

[2]Department of Pharmaceutical Sciences, University of Maryland School of Pharmacy, Baltimore, Maryland 21201, USA.

[3]Stephenson Cancer Center, University of Oklahoma Health Sciences Center, Oklahoma City, Oklahoma 73104, USA.

[4]Harold Hamm Diabetes Center, University of Oklahoma Health Sciences Center, Oklahoma City, Oklahoma, 73104, USA.

*To whom correspondence should be addressed:

Franklin A. Hays

Department of Biochemistry and Molecular Biology

University of Oklahoma Health Sciences Center

Oklahoma City, OK 73104 USA

franklin-hays@ouhsc.edu

Tel: (405) 271-2227 Ext. 61213

Fax: (405) 271-3092

SUPPLEMENTARY FIGURES

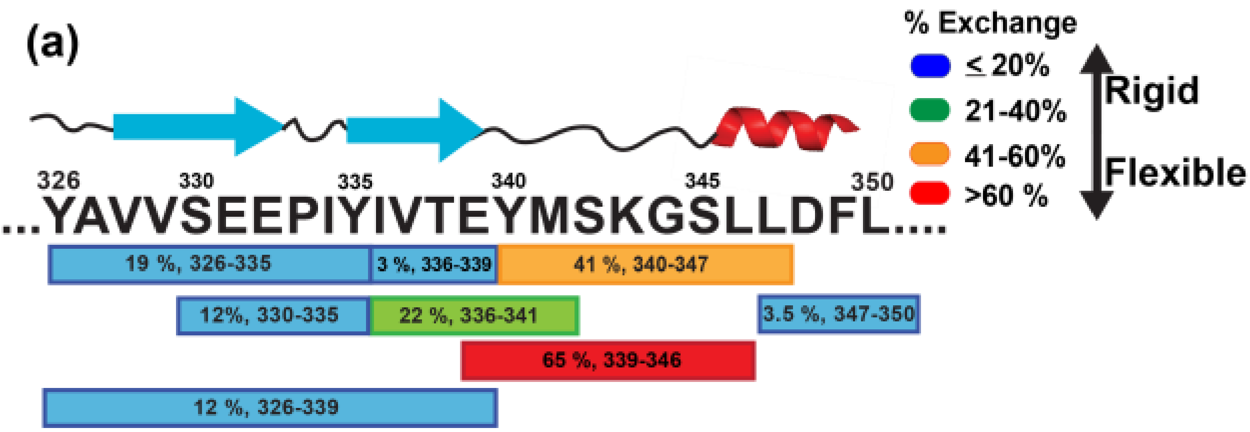

Supplementary Figure 1: Percent peptide exchange derived from the binding cradle region. **(a)** Each peptide is represented in colored bars below the amino acid sequence of binding cradle region. The secondary structures in the region are also indicated as blue arrows (β-strand) and helix cartoon above the amino acid sequence. The color of each bar corresponds to the percent exchange of peptides. The same color classification is used as in the main Figure 2. The percent exchange value and the residue number of each peptide are also indicated.

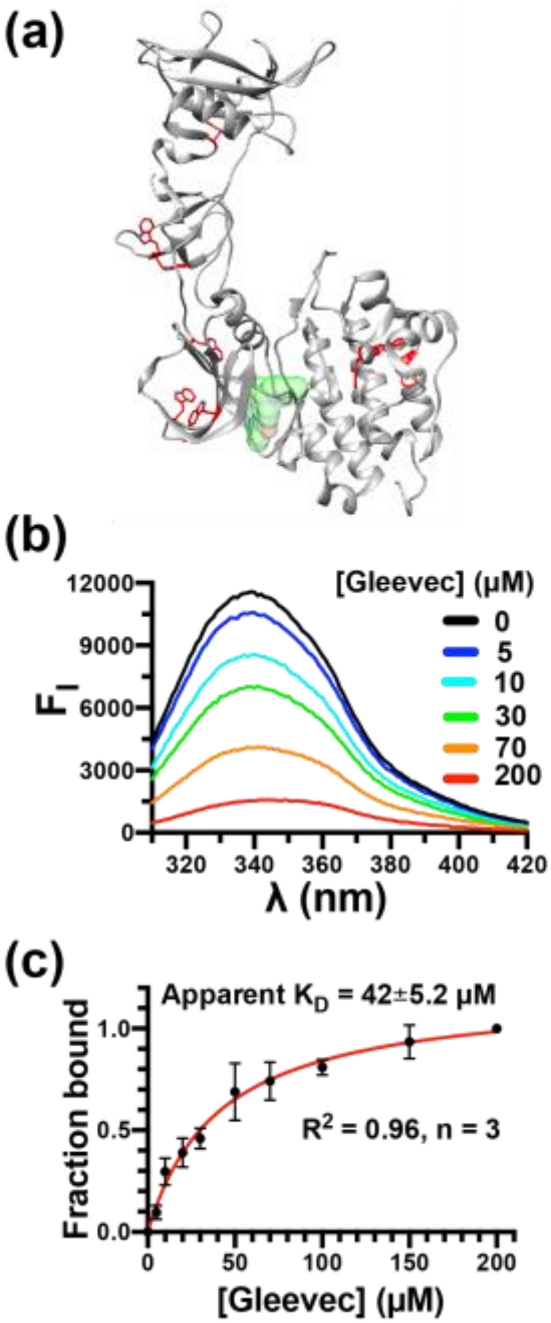

Supplementary Figure 2: Imatinib binding to unphosphorylated c-Src monitored by fluorescence spectroscopy. **(a)** Crystal structure of unphosphorylated c-Src complexed with imatinib (PDBID: 1Y57)[1]. Imatinib is shown in green surface representation. The location of tryptophan residues is shown in red stick. **(b-c)** Imatinib titration to the unphosphorylated c-Src. The intrinsic tryptophan fluorescence of c-Src at different concentrations of imatinib is shown in different colored lines (b). Fraction of c-Src bound to imatinib is plotted against imatinib concentration. The emission maximum at 340 nm in (b) at each imatinib concentration was used to obtain the graph. The data were fitted to a 1:1 binding model.

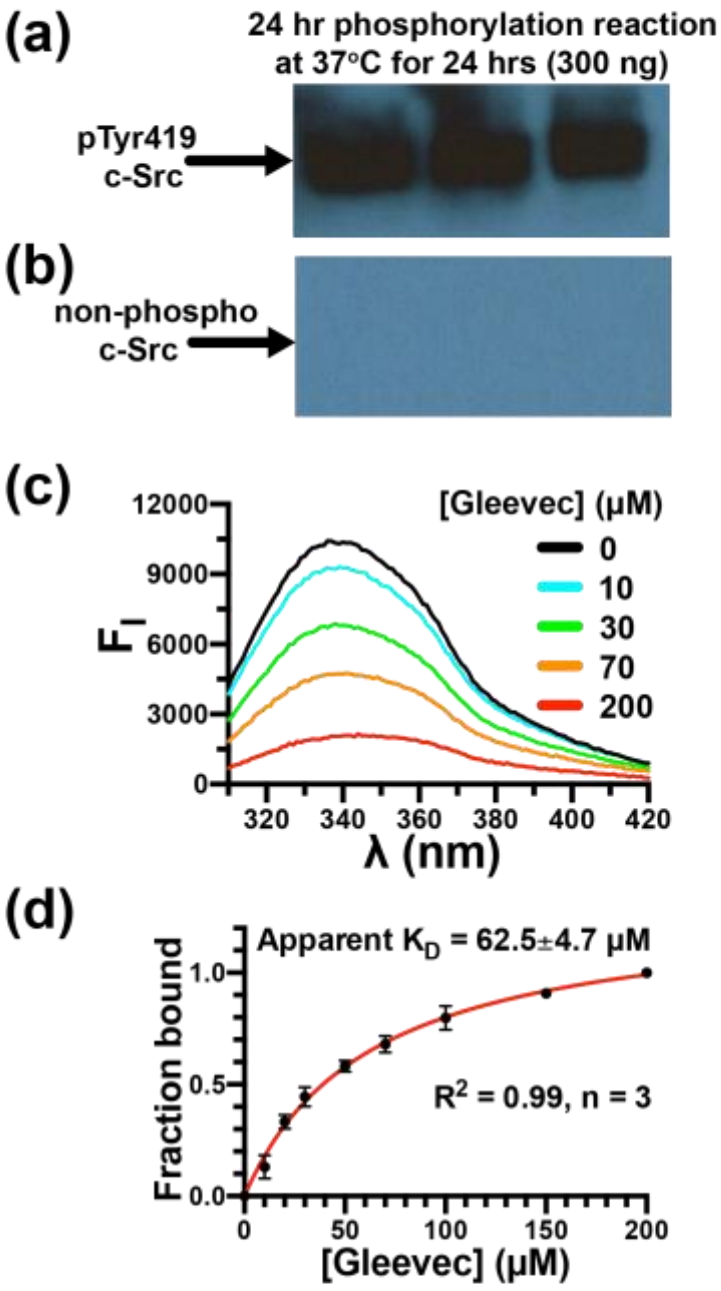

Supplementary Figure 3: Imatinib (Gleevec™) binding to phosphorylated c-Src at Tyr416. **(a-b)** Western blot of c-Src subjected to auto-phosphorylation. (a) Phosphorylated c-Src at Tyr416 was visualized using pTyr416 c-Src specific antibody as described in *Methods*. The same sample was probed with non-phosphorylated c-Src specific antibody to confirm the completion of autophosphorylation reaction. The triplicate samples were shown. **(c-d)** Imatinib titration to pTyr416-c-Src. (c) Imatinib was titrated into the pTyr416-c-Src solution, and the intrinsic tryptophan fluorescence was monitored at indicated imatinib concentration. (d) Fraction of pTyr416-c-Src bound to imatinib is plotted against imatinib concentration. The emission maximum at 340 nm in (c) at each imatinib concentration was used to obtain the graph. The data were fitted to a 1:1 binding model.

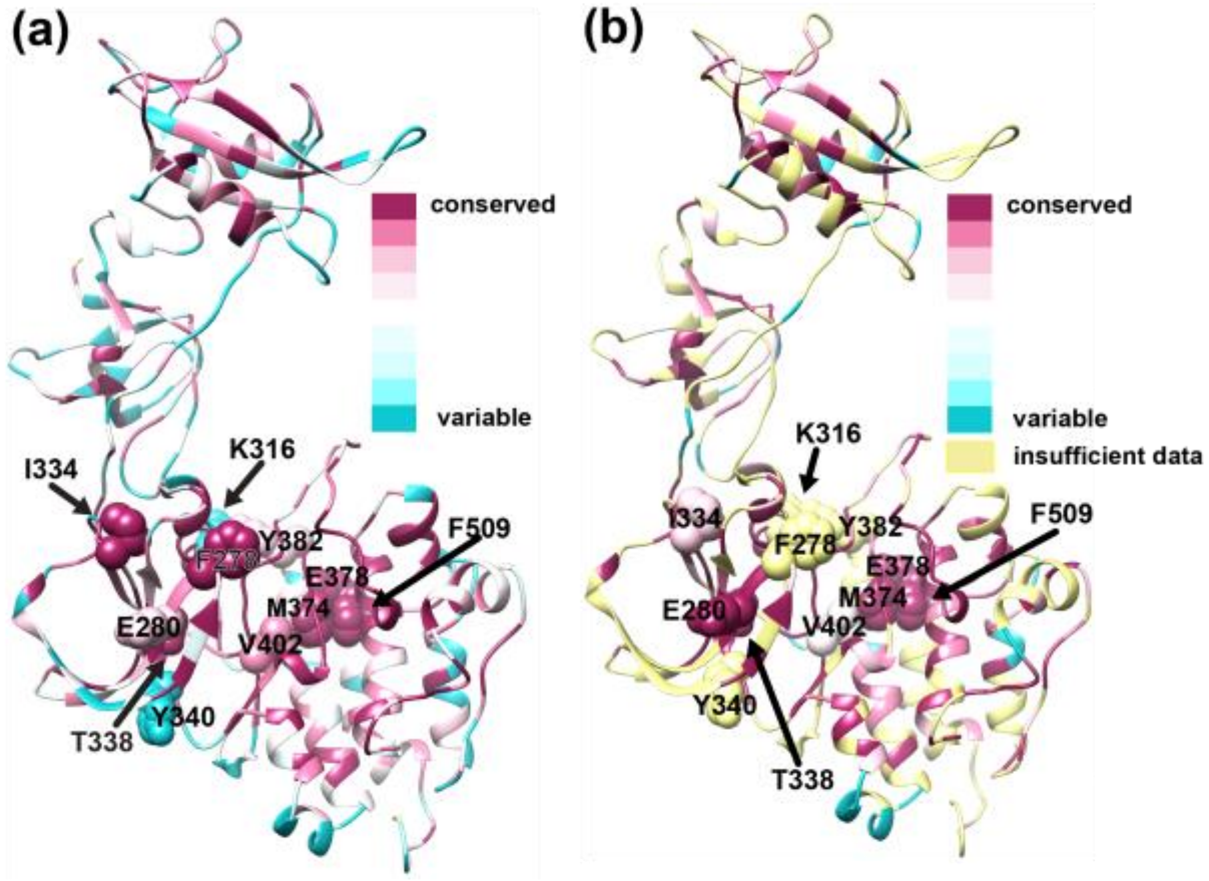

Supplementary Figure 4: Conserved residues in human c-Src. (a) The degree of conservation across all species are highlighted according to the color scheme shown in this figure using Consurf[2]. Clinically identified imatinib resistant mutation sites are labeled and shown in spheres. **(b)** The degree of amino acid sequence similarity in the human Src family. The amino acid sequence of human c-Src, Fyn, Yes, Hck, and Lck was analyzed using Consurf[2].

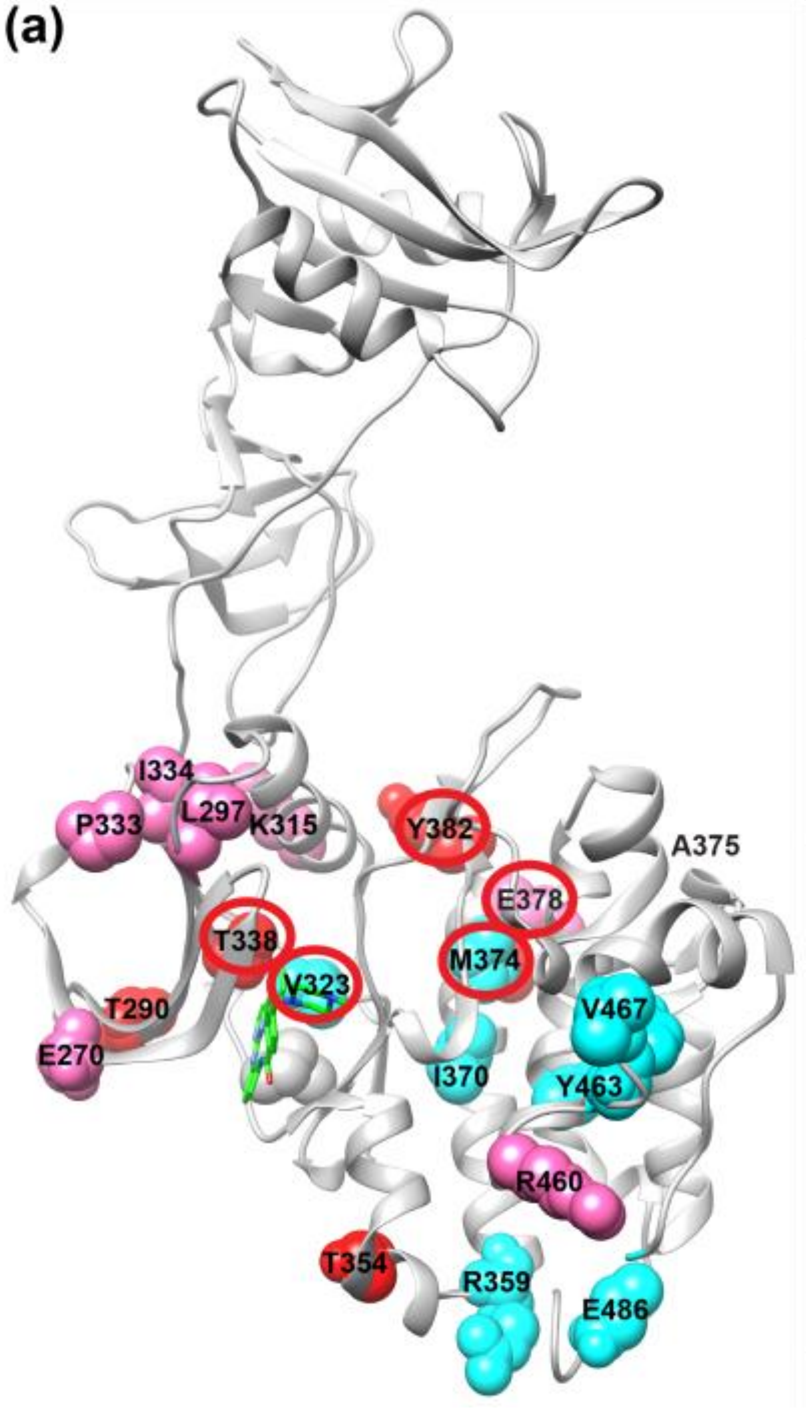

Supplementary Figure 5: Three classes of compound mutation sites. (a) Different classes of compound mutation sites identified in clinical samples[3] are shown in different colors. The seeding mutation sites are indicated in red circles: T338/Y382, M374/V323, or E378. Imatinib resistance-associated compound mutations are labeled and colored according to the seeding mutation sites. Compound mutations appear after one of the seeding mutation sites is mutated.

REFERENCES

1 Cowan-Jacob, S. W. *et al.* The crystal structure of a c-Src complex in an active conformation suggests possible steps in c-Src activation. *Structure* **13**, 861-871 (2005).

2 Landau, M. *et al.* ConSurf 2005: the projection of evolutionary conservation scores of residues on protein structures. *Nucleic Acids Res* **33**, W299-302 (2005).

3 Khorashad, J. S. *et al.* BCR-ABL1 compound mutations in tyrosine kinase inhibitor-resistant CML: frequency and clonal relationships. *Blood* **121**, 489-498 (2013).
